# Gypenoside L and Gypenoside LI Inhibit Proliferation in Renal Cell Carcinoma *via* Regulation of the MAPK and Arachidonic Acid Metabolism Pathways

**DOI:** 10.3389/fphar.2022.820639

**Published:** 2022-03-15

**Authors:** Hui Liu, Xiuming Li, Jinbo Xie, Chengcheng Lv, Fangchao Lian, Shouyi Zhang, Yu Duan, Yu Zeng, Xianglan Piao

**Affiliations:** ^1^ Chengde Medical University, Chengde, China; ^2^ School of Pharmacy, Minzu University of China, Beijing, China; ^3^ Department of Urology, Affiliated Hospital of Chengde Medical University, Hebei, China; ^4^ Department of Urology, Cancer Hospital of China Medical University, Liaoning Cancer Hospital and Institute, Shenyang, China

**Keywords:** gypenoside LI, ccRCC, arachidonic acid, MAPK pathway, gypenoside L

## Abstract

Renal cell carcinoma (RCC) has the highest mortality rate of all urological malignancies. Clear cell renal cell carcinoma (ccRCC) accounts for approximately 80% of all RCC cases and is often accompanied by the accumulation of lipid droplets. Growing evidence indicates that ccRCC is a metabolism-related disease. Gypenosides are commonly used for the clinical treatment of hyperlipidemia, and their antitumor activity has also been recognized. However, the potential inhibitory effects and mechanisms of action of gypenoside L (Gyp L) and gypenoside LI (Gyp LI) in ccRCC remain unclear. In this study, we confirmed that Gyp L and Gyp LI significantly inhibited proliferation and induced apoptosis in ccRCC cells *in vitro*. We performed network pharmacology and RNA-seq, and verified the results by Western blotting, RT-qPCR, and immunofluorescence experiments. Our results demonstrated that Gyp L and Gyp LI upregulate the expression of *COX2* and downregulate the expression levels of cPLA2 and CYP1A1, resulting in reduced arachidonic acid and apoptosis. Gyp L and Gyp LI upregulated the protein levels of DUSP1, p-JUN, and p-JNK, and downregulated p-MEK1/2, p-ERK, and p-P38 levels. Moreover, gypenosides significantly inhibited tumor growth *in vivo*, and gypenosides significantly reduced cPLA2 and CYP1A1 expression. Furthermore, we performed absolute quantification of arachidonic acid (AA) content in ccRCC cells and tumor tissues by HPLC-MS, and found that the arachidonic acid content was significantly reduced after Gyp L, Gyp LI, and gypenoside intervention. In conclusion, our data suggest that Gyp L, Gyp LI, and gypenosides decrease the content of arachidonic acid in ccRCC cells and tumor tissues, but do not have cytotoxic effects on nude mice. Thus, Gyp L, Gyp LI, and total gypenosides extracted from *Gynostemma pentaphyllum* exhibited antitumor activities against ccRCC.

## Introduction

Renal cell carcinoma (RCC) is among the 10 most common cancers worldwide, accounting for 3.7% of all new cancer cases (Siegel et al., 2017). Clear cell renal cell carcinoma (ccRCC), an aggressive cancer originating from the proximal tubular epithelium, accounts for approximately 80% of all cancers (Choueiri and Motzer, 2017). Owing to lipid accumulation, ccRCC cells can be histologically classified by the appearance of clear cytoplasm (Rezende et al., 1999). In addition to the accumulation of intracellular lipid droplets, abnormal fatty acid (FA) metabolism is characteristic of ccRCC cells. Obesity is recognized as a strong risk factor for ccRCC (Renehan et al., 2008; Lowrance et al., 2010; Sawada et al., 2010; Keum et al., 2015).

Inflammation in the tumor microenvironment (TME) is a major factor driving tumor progression and is one of the hallmarks of cancer, while eicosanoids have been closely linked to inflammation and cancer (Greene et al., 2011). AA and eicosanoids play a central role in many diseases, including cancer and obesity (Wang and Dubois, 2010; Dennis and Norris, 2015; Sonnweber et al., 2018). Previous studies have shown that PLA2s is the initial enzyme of the AA metabolic pathway that converts membrane-bound arachidonyl phospholipids under several stimuli to form free fatty acids, predominantly AA and LPLs (Dessen et al., 1999; Gijón and Leslie, 1999; Yarla et al., 2016a). Key enzymes in the AA metabolic pathway include cyclooxygenase (COX), lipoxygenase (LOX), and cytochrome P450 (CYP). Furthermore, prostaglandins (PGs), leukotrienes (LTs), epoxy/hydroxy-eicosatrienoic acids, and other bioactive signaling oxylipids play key roles in the treatment of inflammation and cancer (Wang and Dubois, 2010; Yarla et al., 2016b). The relationship between AA and PI3K (Hughes-Fulford et al., 2006) and the MAPK signaling pathway has previously been reported (Alexander et al., 2006). However, the cancer-associated signaling pathways and the relationship between these bioactive lipids and cell proliferation remain largely unclear.

*Gynostemma pentaphyllum* (*G. pentaphyllum*) is commonly used as a source of medicine in China and Southeast Asia, in the treatment of various diseases, including hyperlipidemia and tumors. Gypenoside LVI is a monomer compound of *G. pentaphyllum*, which can be used to treat atherosclerotic cardiovascular disease (ASCVD) by increasing LDL-C uptake in hepatocytes by inhibiting PCSK9 expression (Wang et al., 2021). *G. pentaphyllum* exerts anti-hyperlipidemic effects by reducing triglycerides, cholesterol, and nitrite (Megalli et al., 2005). The antihyperlipidemic mechanisms of gypenosides may regulate lipid metabolism disorders and ameliorate hepatic function (Yang et al., 2013). Previous studies have reported that Gyp L and Gyp LI, dammarane-type saponins from *G. pentaphyllum*, induced apoptosis and inhibit proliferation in a variety of cancers, including esophageal cancer, hepatocellular carcinoma, breast cancer, melanoma, and lung cancer (Zheng et al., 2016; Xing et al., 2018; Ma et al., 2019; Zu et al., 2020; Zu et al., 2021). Importantly, our previous investigations have reported that the total saponins of *G. pentaphyllum* induced apoptosis in ccRCC by regulating the PI3K/AKT/mTOR pathway *in vitro*. However, the effects and mechanisms of the monomer compounds Gyp L and Gyp LI, individual dammarane-type saponins isolated from steamed *G. pentaphyllum* have not yet been explored in RCC. Based on previous findings, we hypothesized that Gyp L and Gyp LI could induce apoptosis in ccRCC, and thus, we investigated the effect of gypenoside L and gypenoside LI on apoptosis.

## Materials

### Chemicals and Reagents

The extraction and identification of gypenoside L (Gyp L) and gypenoside LI (Gyp LI) were performed as previously described (Xing et al., 2018). The purity of Gyp L and Gyp LI was determined to be >99% using liquid chromatography-mass spectrometry (LC-MS). The antibodies used included Anti-Cytochrome C (ab13575); Bcl-2 (124) (15071S; Cell Signalling Technology, Inc.), Bax (2772S; Cell Signalling Technology, Inc.), cyclin A (sc-271682; SANTA), CDK2 (sc-6248; SANTA), CDK1 (ab245318; Abcam), cyclin B1 (sc-8396; SANTA), and JNK antibody (#9252; Cell Signalling Technology, Inc.); Phospho-JNK (Thr183/Tyr185) (81E11; Cell Signalling Technology, Inc.), c-Jun (60A8; #9165; Cell Signalling Technology, Inc.), Phospho-c-Jun (Ser73) (D47G9; #3270; Cell Signalling Technology, Inc.), P44/42MAPK (Erk1/2) (137F5; Cell Signalling Technology, Inc.), Phospho-p44/42 MAPK(Erk1/2) (20G11; Cell Signalling Technology, Inc.), P38 MAPK (#9212; Cell Signalling Technology, Inc.), p-p38 MAPK (D-8) (sc-7973; SANTA), MKP-1 (E-6) (sc-373841; SANTA), cPLA2 (#2832, Cell Signalling Technology, Inc.), LOX-1 (ab60178; Abcam), COX-2 (H-3; sc-376861; Santa Cruz Biotechnology), and CYP1A1(A-9) (sc-393979; SANTA Cruz).

### Cell Culture

The human ccRCC cell line ACHN was cultured in DMEM (Gibco, China), while 769-P cells were cultured in RPMI 1640 medium (Gibco, China), supplemented with 10% fetal bovine serum (FBS, BioInd) and 1% penicillin–streptomycin (BasalMedia). ACHN and 769-P were maintained at 37°C and 5% CO_2_.

### CCK8 Assay

For the Gyp L and Gyp LI viability assays, 1 × 10^4^ ACHN or 769-P cells were seeded in 96-well plates and allowed to adhere for 12 h. DMSO or 20–100 μM Gyp L/Gyp LI were added to the wells after washing twice with PBS. Cell viability was detected after 48 h by CCK8 assay (Dojindo Molecular Technologies, Inc.), and the absorbance was measured at 450 nm using a microplate reader (BMG Labtech FLUOstar Omega).

### Colony Formation Assays

For colony formation assays, 5 × 10^2^ ACHN and 769-P single cells were seeded in 12-well plates. Wells were washed twice with PBS after being allowed to adhere for 12 h and supplemented with either 0.1% DMSO, Gyp L, or Gyp LI. Colonies were stained with 0.1% crystal violet after 14 days and quantified using ImageJ software.

### Cell Apoptosis Assays

To evaluate cell apoptosis, 1 × 10^5^ ACHN or 769-P cells were seeded in 12-well plates and treated with 0.1% DMSO, Gyp L, or Gyp LI for 48 h. The cells were detached using trypsin, washed with cold PBS, and resuspended in 1× binding buffer. The cells were incubated for 20 min with 5 μl of FITC and 5 μl of PI for 5 min in the dark. Apoptosis was measured using a FACSCalibur flow cytometer (BD FACSDiva 8.0.1.1), and the data were analyzed using FlowJo_V10 software.

### Cell Cycle Analysis

For cell cycle analysis, 2 × 10^5^ ACHN or 769-P cells were seeded in six-well plates and treated with DMSO, Gyp L, or Gyp LI for 48 h. Wells were then washed with ice-cold PBS, the cells were trypsinized, and the cell suspensions were fixed in cold 70% ethanol at 4°C for 24 h. Furthermore, cells were stained with propidium iodide (PI) and incubated at 37°C for 30 min. The cell cycle distribution was subsequently analyzed using BD FACSDiva 8.0.1.1, and the data were analyzed using the ModfitLT 5 software.

### Collation of Targets for Gypenoside L, Gypenoside LI, and Renal Cell Carcinoma

The targets of gypenoside L and gypenoside LI were retrieved from the Traditional Chinese Medicine Database and Analysis Platform (TCMSP) (https://tcmsp-e.com/), and RCC-related genes were searched from three databases: TTD (http://db.idrblab.net/ttd/) (Zhou et al., 2021), Online Mendelian Inheritance in Man (OMIM) (https://www.omim.org/), and The Human Gene Database (Genecards) (https://www.genecards.org/).

### Gene Ontology and KEGG Analysis

Gene ontology (GO) and KEGG pathway analyses were performed using the DAVID Bioinformatics Resources ver. 6.8 (https://david.ncifcrf.gov/) (Huang da et al., 2009). Functional annotation clustering was used to select terms that met the cutoff limits of count ≥2, EASE scores ≤0.05, and *p* < 0.05.

### RNA-Seq

To evaluate the effects of Gyp L and Gyp LI on the mRNA levels of 769-P and ACHN cells, we obtained RNA from cells after 48 h of treatment with Gyp L and Gyp LI. The concentration of Gyp LI was 45 and 55 μM for 769-P and ACHN, respectively, while the concentrations of Gyp L in 769-P and ACHN cells were 60 and 70 μM, respectively. TRIzol Total RNA Isolation Kit (Tiangen, Beijing, China) was used to isolate total RNA from ACHN and 769-P cells. RNA-seq was performed by Qinglian Biotech Corporation (Beijing, China). Briefly, RNA samples were sequenced on the Illumina HiseqX10 platform and analyzed using DAVID Bioinformatics Resources6.8 (https://david.ncifcrf.gov/) and Metascape (http://metascape.org/gp/index.html#/main/step1).

### Immunofluorescence

For immunofluorescence assays, cells were fixed with 4% paraformaldehyde at room temperature for 15 min, permeabilized with 0.3% Triton X-100 for 20 min, and then blocked with 2% BSA/PBS for 1 h. After blocking, the cells and cPLA2 primary antibodies were incubated overnight at 4°C in a humidified box. The next day, cells were incubated with fluorescently labeled secondary antibodies for 1 h, the nuclei were stained with DAPI (Beyotime, China), and images were captured using an Olympus microscope.

### Quantitative RT-PCR

RNA was extracted from cells using the TRIzol reagent kit (TIANGEN BIOTECH Co., Ltd.). cDNA was synthesized from the total RNA using PrimeScript^™^ RT Master Mix CFX96 Real-Time System (Bio-Rad). The primer sequences used in this study were as follows: PLA2G4, forward: 5′-AAT​ACT​GCA​CAA​TGC​CCT​T TACC-3′, reverse: 5′-GCT​TCC​AAA​TAA​GTC​GGG​AGC-3′. COX7A, forward: 5′-CCA​AAT​GCT​TTA​CCG​GAC​CAC-3′, reverse: 5′-GCTGCGAAGCCATG TAGAG G-3′. ALOX5, forward: 5′-CTC​AAG​CAA​CAC​CGA​CGT​AAA-3′, reverse: 5′-CCT​TGT​GGC​ATT​TGG​CAT​CG-3′. CYP2E1, forward: 5′-GGGAAACAGGGCA ATGAGAG-3′, reverse: 5′-GGA​AGG​TGG​GGT​CGA​AAG​G-3′. c-FOS, forward: 5′-CAG​GCG​GAG​ACT​GAC​AA ACTG-3′, reverse: 5′-TCC​TTC​CGG​GAT​TTT​GC AGAT-3′. JUN, forward: 5′-GGATATTGGAT TCCGACTCGAC-3′, reverse: 5′-GGG ATC​AAG​TAG​CTC​AAT​CAG​C-3′. DUSP1, forward: 5′-AGGTGGGTTTGCTGAG TTCTC-3′, reverse: 5′-CTCGGGGATAAAGTC AGGCTT-3′. GAPDH, forward: 5′-GGA​GCG​AGA​TCC​CTC​CAA​AAT-3′, reverse: 5′-GGC​TGT​TGT​CAT​ACT​TCT​CA TGG-3′. ChamQ^™^ SYBR Color qPCR Master Mix (Vazyme) was used for the reaction. Briefly, the cycling conditions were as follows: predenaturation for 60 s at 95°C, followed by 45 cycles of 10 s at 95°C (denaturation) and 30 s at 60°C (annealing).

### Immunoblot Analysis

769-P and ACHN cells were lysed using RIPA buffer (Beyotime). Subsequently, the lysate was centrifuged at 12,000 × *g* for 30 min at 4°C, and concentrations were quantified using a BCA assay kit (Beyotime Biotechnology, Jiangsu, China). Lysates were boiled at 99°C for 5 min, separated by SDS-PAGE electrophoresis using 10% gels, and transferred to a polyvinylidene fluoride (PVDF) membrane at 250 mA for 2 h. The membrane was incubated with the primary antibody at 4°C overnight after blocking with 5% skim milk for 1 h. All primary antibodies were used in a 5% BSA/TBST solution at the dilutions indicated in the instructions of the manufacturers. The membranes were then incubated with a secondary antibody at a dilution of 1:5,000 in 5% milk.

### Lipid Extraction

Total lipids were extracted based on the method of Koundouros et al. (2020). In brief, the media of 769-P and ACHN cells grown to 80% confluency were replaced with FBS-free medium, cells were cultured for 1 h, and then lipids were extracted. For extraction, cells were collected and resuspended in 1 ml of water, then 3.75 ml of chloroform/methanol mixture (1:2 v/v) was added, and the samples were vortexed and incubated on ice for at least 30 min. Afterward, 1.25 ml of chloroform was added followed by 1.25 ml of water. The samples were then vortexed and centrifuged at 1,000 rpm at 4°C for 10 min, and the organic bottom phase was separated and dried under a nitrogen flow for analysis.

### Xenograft

ACHN cells (5 × 10^6^) were subcutaneously inoculated into both flanks of 4- to 6-week-old male immunodeficient BALB/c nude mice weighing 14–16 g. Either a normal diet or 100 mg/kg of gypenosides was provided daily through oral gavage for 21 days when tumors reached a volume of 100–200 mm^3^. After 3 weeks, the mice were sacrificed, and the tumor tissues were subjected to immunohistochemical detection and arachidonic acid metabolism. All animal experiments were approved by the Animal Research Ethics Committee of China Medical University.

### Immunohistochemistry

To assess the expression of Ki-67 *in vivo*, fresh frozen tumor pieces were sectioned to a thickness of 10 mM and boiled in citrate unmasking solution for 35 min. After incubation in 3% hydrogen peroxide for 10 min, the sections were blocked with 5% BSA for 1 h at room temperature, and then with rabbit anti-human Ki-67 (1:100, Abcam, United States) antibody overnight at 4°C. Next, the slides were incubated with an appropriate secondary antibody (Zsbio, China), and stained with DAB and hematoxylin.

### Statistics

All statistical analyses were performed using SPSS 25.0 and GraphPad Prism 8. All data are presented as the mean ± SD, and analyzed using a variance (ANOVA). Significance was defined as *p* < 0.05, and all experiments were performed at least three times.

## Results

### Antiproliferation Activity of Gypenoside L and Gypenoside LI in Clear Cell Renal Cell Carcinoma Cells

**FIGURE 1:**
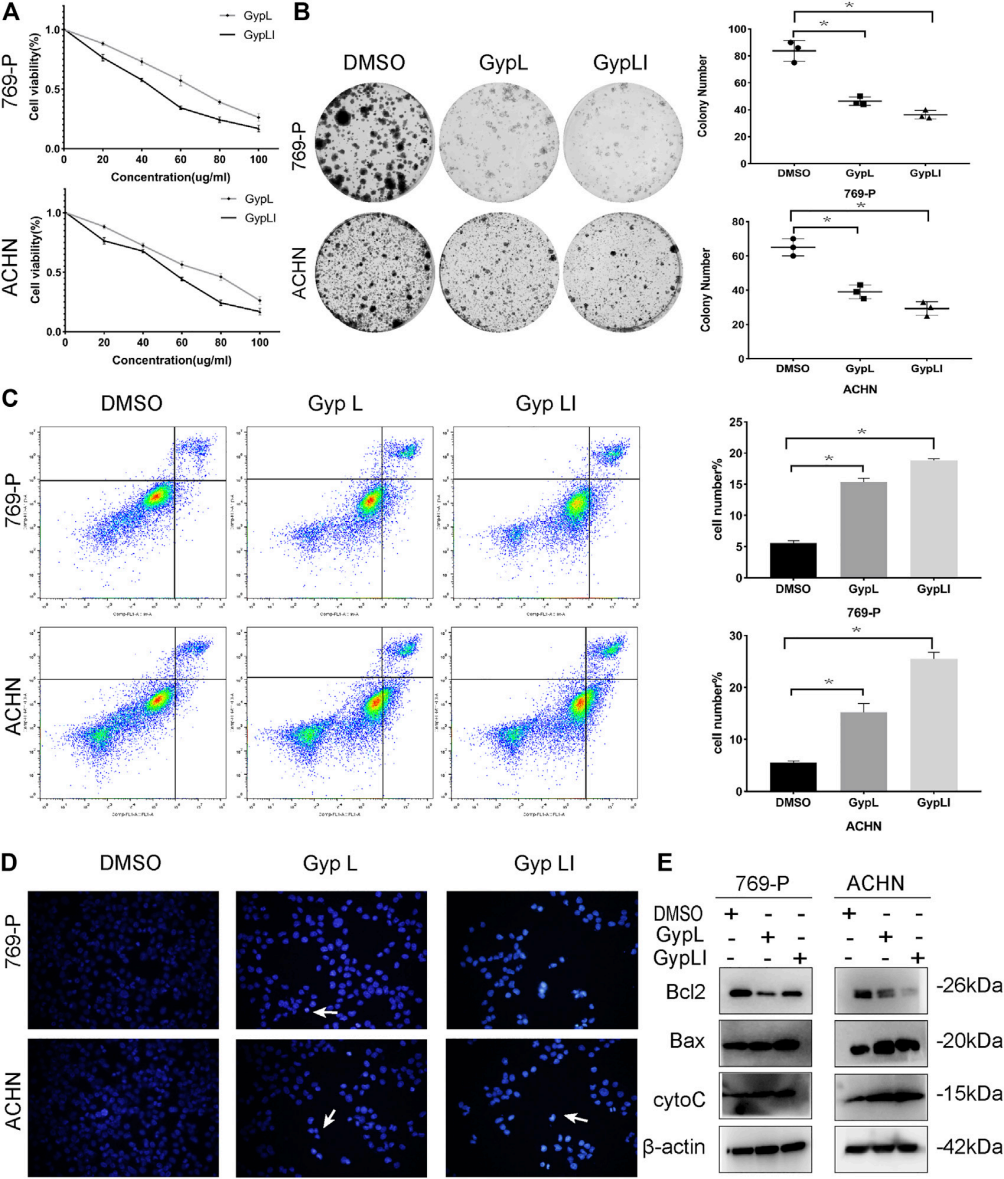
Gypenoside L (Gyp L) and gypenoside LI (Gyp LI) inhibit proliferation and induce apoptosis of clear cell renal cell carcinoma (ccRCC) cells. **(A)** CCK8 assays were performed to detect the cell viability of 769-P and ACHN cells after different doses of Gyp L and Gyp LI treatment for 48 h. **(B)** The Colony formation assays were used to detect clonogenicity of 769-P and ACHN cells after Gyp L and Gyp LI treatment. **(C)** The apoptosis of 769-P and ACHN cells treated with Gyp L and Gyp LI was analyzed *via* flow cytometry. **(D)** Hoechst 33258 was used for ACHN and 769-P cell apoptosis detection, stained, and observed with a fluorescence microscope. **(E)** The protein levels of Bax, Bcl2, and cytochrome C were detected by Western blotting experiments. Data are presented as the mean ± SD of expression levels from three independent experiments (*p < 0.05, vs. control group).

To ascertain the inhibitory effects of Gyp L and Gyp LI on renal cancer cells, we conducted CCK8 assays on ACHN and 769-P cells after 48 h of treatment with Gyp L and Gyp LI at different concentrations (0, 20, 40, 60, 80, and 100 μM). We observed that Gyp L can significantly inhibit the viability of ccRCC cells, with half-maximal inhibitory concentrations (IC_50_) of 60 and 70 μM in 769-P and ACHN cells, respectively. The inhibitory effect of Gyp LI on ccRCC cells was stronger than that of Gyp L, with IC_50_ values of 45 and 55 μM for 769-P and ACHN, respectively. These results show that both Gyp L and Gyp LI significantly inhibit cell viability in a dose-dependent manner (Figure 1A).

In addition to assessing viability, we demonstrated that Gyp L and Gyp LI significantly reduced the clonogenicity of 769-P and ACHN cells (Figure 1B). Furthermore, we evaluated the effects of Gyp L and Gyp LI on apoptosis in the two cell lines by flow cytometry and Hoechst 33258 assays. As shown in Figures 1C, D, after Gyp L and Gyp LI treatment, the apoptosis rate of the two cell lines was significantly increased. Furthermore, we evaluated the expression of the apoptosis-related proteins Bax, Bcl2, and cytochrome C by Western blotting. Both Gyp L and Gyp LI downregulated the expression of the apoptosis-inhibiting protein Bcl2 and upregulated the expression of Bax and cytoC (Figure 1E). These results indicate that Gyp L and Gyp LI inhibit cell proliferation and induce apoptosis in ccRCC cells.

### Gypenoside L and Gypenoside LI induced Cell cycle arrest in Clear Cell Renal Cell Carcinoma Cells

**FIGURE 2:**
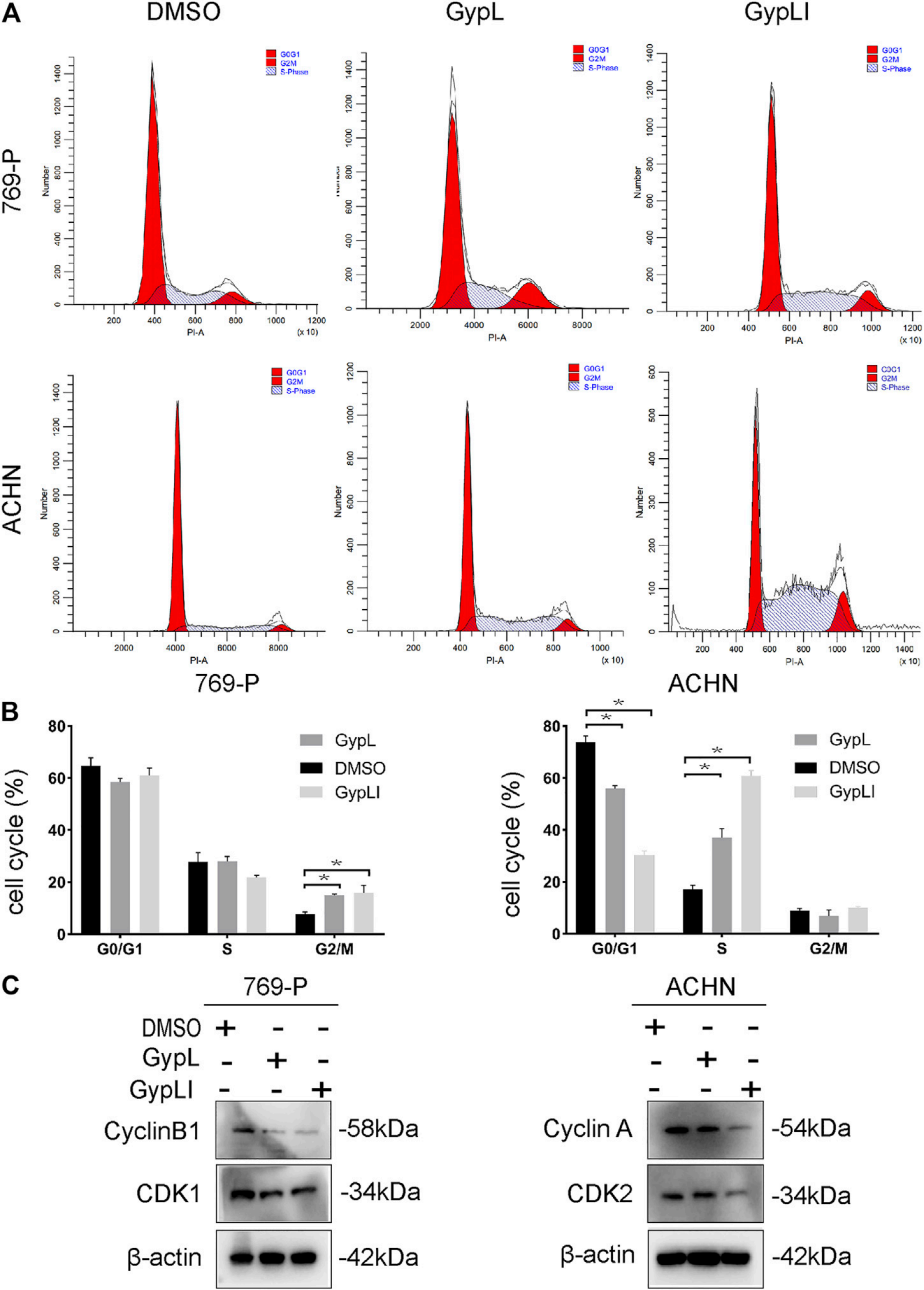
Gyp L and Gyp LI induced cell cycle arrest in 769-P and ACHN cells. **(A)** Gyp L and Gyp LI arrest cell cycle at G2/M phase in 769-P, while G1/S phase arrest after Gyp L and Gyp LI treatment in ACHN, were determined by flow cytometry. **(B)** Statistical graph of three independent repeated experiments. **(C)** The protein levels of cyclin A and B1, CDK1, and CDK2 were detected by Western blotting. Data are expressed as the mean± SD of expression levels from three independent experiments (*p < 0.05, vs. control group).

To detect the effect of Gyp L and Gyp LI on the cycle distribution of ccRCC cells, cell cycle analysis was performed for 769-P and ACHN cells using flow cytometry. The results showed that Gyp L and Gyp LI treatment blocked 769-P cells in the G2/M phase. In ACHN cells, after treatment with Gyp L and LI, the cells were arrested in the G1/S phase (Figures 2A,B). Furthermore, Western blotting revealed that the expression levels of cyclin A and B1, CDK1, and CDK2 were all reduced after treatment with Gyp L and Gyp LI (Figure 2C).

### Network Pharmacology and Transcriptomics to Predict Key Targets and Pathways

**FIGURE 3:**
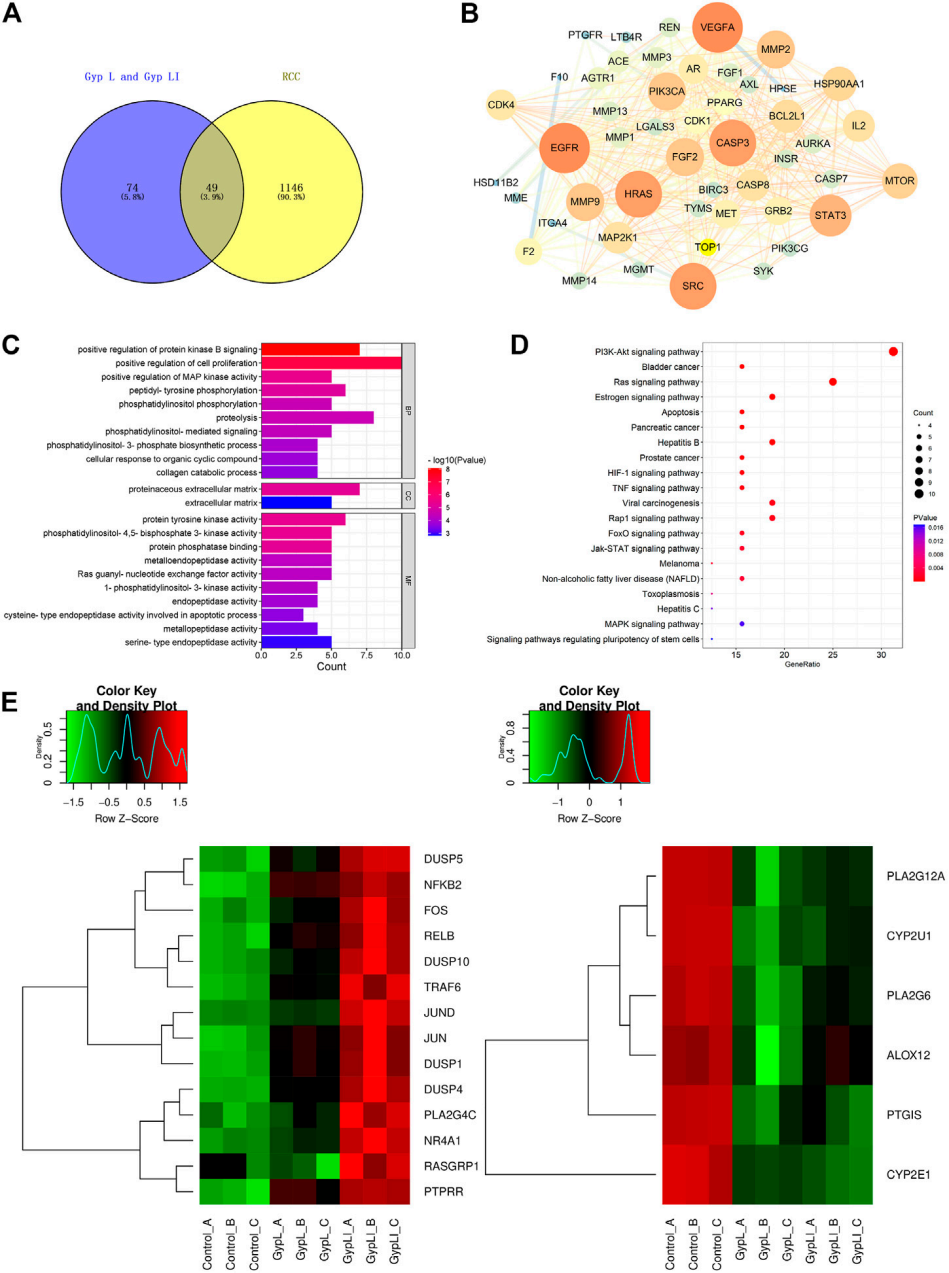
Analyses of the key targets and pathways of Gyp L and Gyp LI inhibiting ccRCC cells through network pharmacology and RNA-seq. **(A)** Venn diagram summarizing the intersection targets between Gyp L, Gyp LI, and renal cell carcinoma (RCC). **(B)** Protein interaction network diagram of 49 intersection targets was constructed through the cytoscape platform. **(C)** Gene ontology (GO) analysis of 49 targets in terms of biological processes, cell components, and molecular functions. **(D)** The KEGG pathway that Gyp L and Gyp LI affect the RCC process analyzed by R language. **(E**, **F)** Unsupervised hierarchical clustering of mitogen-activated protein kinase (MAPK) **(E**,**F)** arachidonic acid.

Using network pharmacology to study the molecular mechanisms of actions of these drugs, we first examined whether the target genes of Gyp L and Gyp LI correlated with genes involved in RCC. For this, we screened 123 targets of Gyp L and Gyp LI using the Swiss target prediction platform, and further screened 1,195 renal cancer-related target genes using the TTD, OMIM, and Genecards databases. These targets were then crossed on the VENNY platform, and 49 common genes were obtained for further analyses (Figure 3A). Furthermore, we constructed a protein interaction network of 49 overlapping genes to predict the hub genes obtained using the Cytoscape platform (Figure 3B). To identify the biological characteristics of Gyp L and Gyp LI on RCC, GO and pathway enrichment analyses were performed using DAVID 6.8. Forty-one biological processes (BP), four cell components (CC), and 25 molecular function (MF) terms met the requirements of Count ≥ 2 and EASE score ≤ 0.05. Detailed GO information is shown in Supplementary Table S2. The first 22 significant terms in the BP, CC, and MF categories are shown in Figure 3C. Of note, GO enrichment analysis revealed that the related biological functions mainly include enzyme activation, cell proliferation, and other functions. To explore the target pathways of Gyp L and Gyp LI in RCC, KEGG analysis of common targets was performed. The pathway information of Gyp L and Gyp LI on RCC is shown in Supplementary Table S1. The top 20 significant pathways of Gyp L and Gyp LI on RCC are shown in Figure 3D, ranking hits according to the *p*-value, from most to least significant. KEGG pathway enrichment identified the PI3K/AKT and Ras/MAPK pathways as key. To shed light on the mechanism by which Gyp L and Gyp LI act on ccRCC cells, we performed transcriptome sequencing analysis of 769-P and ACHN cells treated with Gyp L and Gyp LI. We compared the transcriptomes of Gyp L-/Gyp LI-treated cells vs. Control, and found that genes related to the MAPK pathway, such as DUSP1, FOS, c-JUN, etc., were significantly upregulated (log2 fold change >1, *p* < 0.05), while the enzymes related to the arachidonic acid metabolism pathway, including PLA2G4, COX7A, ALOX12, and CYP2E1, were significantly downregulated (log2 fold change < −1, *p* < 0.05) (Figures 3E, F) (Supplementary Tables S4, S5). Arachidonic acid is an omega-6 polyunsaturated fatty acid. Its metabolism-related enzymes and metabolites participate in inflammation and regulate a variety of cellular processes, including cell proliferation, angiogenesis, tumor invasion, and metastasis (Wang and Dubois, 2010). Based on the results of our network pharmacology and RNA-seq analyses, we speculated that Gyp L and Gyp LI inhibit ccRCC cells by modulation of the MAPK pathway. Both *G. pentaphyllum* and RCC have previously been strongly linked to lipid metabolism; thus, in subsequent experiments, we focused on arachidonic acid metabolism.

### Gypenoside L and Gypenoside LI Act on Tumor Cells *via* the Mitogen-Activated Protein Kinase and Arachidonic Acid Metabolism Regulatory Mechanisms

**FIGURE 4:**
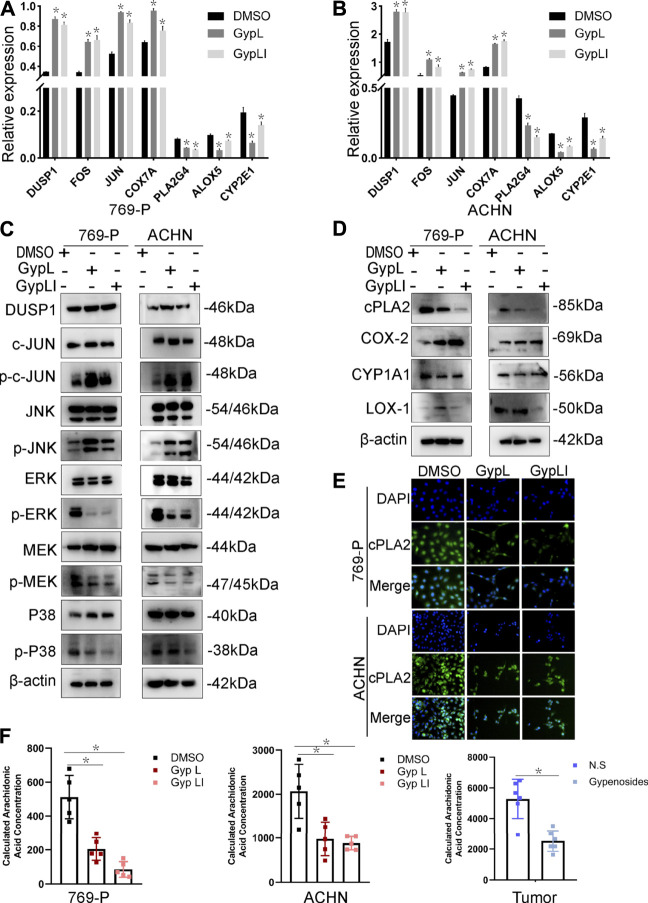
Expression of MAPK and arachidonic acid metabolism signaling-related genes and the reduced content of AA after Gyp L and Gyp LI treatments. **(A**, **B)** The expressions of arachidonic acid metabolism signaling-related genes COX7A, PLA2G4, ALOX5, CYP2E1 and MAPK pathway-related genes DUSP1, FOS, JUN in 769-P (A) and ACHN (B) cells were detected by RT-qPCR. **(C)** The expression of MAPK-related proteins after Gyp L and Gyp LI treatments were detected *via* Western blotting. **(D)** The expression of arachidonic acid metabolism signaling-related proteins after Gyp L and Gyp LI treatments were detected *via* Western blotting. **(E)** The expression of cPLA2 after Gyp L and Gyp LI treatment was detected by immunofluorescence assays. **(F)** The levels of arachidonic acid in 769-P and ACHN treated with Gyp L and Gyp LI and in tumor tissues after gypenoside treatment were measured by liquid chromatography-mass spectrometry (LC/MS). Data are presented as the mean ± SD of expression levels from three independent experiments (*p < 0.05, vs. control group).

To further confirm the network pharmacology and RNA-seq results, and to ascertain the mechanisms by which Gyp L and Gyp LI inhibit tumorigenesis in ccRCC cells, we evaluated the effects of the two drugs on MAPK and arachidonic acid pathway-related genes in ACHN and 769-P lines. Furthermore, we performed RT-qPCR to detect key genes involved in the MAPK and arachidonic acid pathways. We observed that Gyp L and Gyp LI significantly upregulated the expression of DUSP1, FOS, JUN, and COX7A, while downregulating the expression of PLA2G4, ALOX5, and CYP2E1 (Figures 4A,B). Through Western blotting (WB) analysis, we found that the protein levels of DUSP1, p-JUN, and p-JNK were upregulated in 769-P and ACHN cells, while the p-MEK1/2, p-ERK, and p-P38 levels were downregulated after Gyp L and Gyp LI intervention (Figure 4C). The PI3K and MAPK pathways regulate arachidonic acid metabolism *via* the cPLA2 kinase (Koundouros et al., 2020). Therefore, we further detected the expression levels of the enzymes in the arachidonic acid metabolism pathway using WB, revealing that Gyp L and Gyp LI upregulated COX-2 and simultaneously downregulated the expression of CYP1A1 and cPLA2 (Figure 4D). The immunofluorescence results were consistent with the RT-qPCR and WB results. Gyp L and Gyp LI reduced the cPLA2 levels in 769-P and ACHN cells (Figure 4E). cPLA2 is the initial enzyme involved in arachidonic acid metabolism, which promotes the release of AA (Wang and Dubois, 2010). AA levels were significantly reduced when cPLA2 was inhibited. Interestingly, UHPLC-MS profiling analysis revealed that substantial AA reduction was observed in xenograft tumors and cells following Gyp L and Gyp LI treatment, compared with the control group (Figure 4F).

### Gypenoside L and Gypenoside LI Inhibit Tumor Growth by Reducing the Arachidonic Acid Content

**FIGURE 5:**
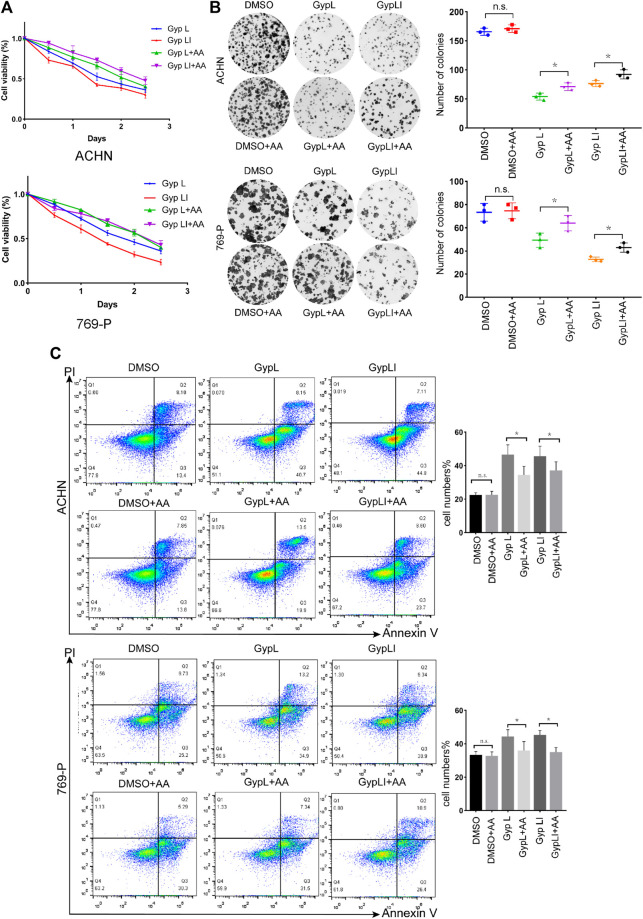
The supplement of AA rescued the killing effect of Gyp L and LI. **(A)** Cell viability of 769-P and ACHN cells following treatment with different doses of Gyp L and Gyp LI for 48 h, supplemented with or without AA. **(B)** Clonogenic assays of 769-P and ACHN cells treated with different doses of Gyp L and Gyp LI under conditions, supplemented with or without AA. **(C)** Cell apoptosis of 769-P and ACHN after treatment with Gyp L and LI, with or without AA detected by flow cytometry. Data are presented as the mean ± SD of expression levels from three independent experiments (n.s., not significant; *p < 0.05, vs. control group).

We designed a rescue experiment to further study the mechanism of action of Gyp L and Gyp LI on 769-P and ACHN cells. Cell medium was supplemented with AA and treated with Gyp L and LI. Through the CCK8 assay, we observed that supplementation with AA rescued the killing effect of Gyp L and Gyp LI (Figure 5A). We further used clone formation experiments to confirm the importance of arachidonic acid supplementation on the cloning ability of Gyp L and Gyp LI in ccRCC cells. We observed a marked reduction in the number of colonies following Gyp L and Gyp LI treatment in 769-P and ACHN cells, which was restored in the presence of AA (Figure 5B). In addition, flow cytometry was used to detect the effect of arachidonic acid supplementation on the apoptosis of ccRCC cells induced by Gyp L and Gyp LI. The results revealed that supplementation with arachidonic acid reversed the effects of Gyp L and Gyp LI on the induction of ccRCC cell apoptosis (Figure 5C).

### Antitumour Effects of Gypenosides on Tumor Growth in Vivo

**FIGURE 6:**
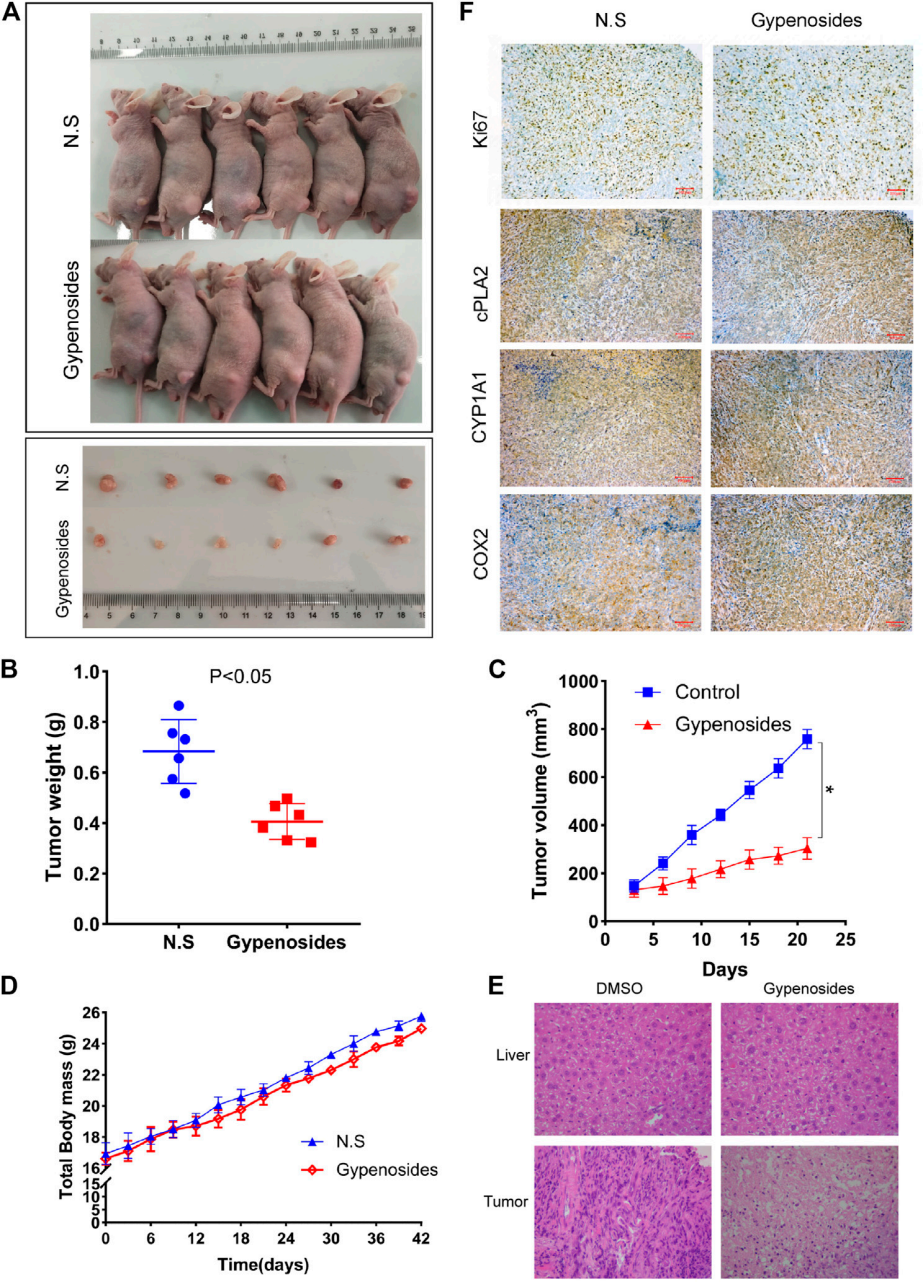
Gypenosides suppressed the growth of RCC cell xenograft tumors *in vivo*. **(A)** Nude mice were treated with saline or gypenosides for 28 days, and pictures of nude mice and tumors were obtained. **(B)** Changes in tumor weight after gypenoside administration, compared with the control group. **(C)** Changes in tumor volume after administration of gypenosides, compared with the control group. **(D)** The body weight of the two groups of mice during the entire experiment. **(E)** HE staining of mice liver and tumors. **(F)** The differences in the protein expression levels of Ki67, cPLA2, CYP1A1, and COX2 between the two tumor groups were detected by immunohistochemistry.

It is known that Gyp L, Gyp LI, and gypenosides can inhibit the proliferation of ccRCC cells *in vitro* (Liu et al., 2021). We further confirmed whether gypenosides could inhibit tumor growth *in vivo*. As shown in Figures 6A, C, gypenoside-treated ACHN cell xenografts grew much slower than those of the control group. Consistently, the weight of tumors was 37% lower, on average, after treatment with gypenosides compared with tumors from control mice (Figure 6B). However, there was almost no difference in the body weights of the two groups of mice during the entire experiment (Figure 6D). HE staining analysis revealed that the livers of mice treated with gypenosides showed no significant difference compared with the control group, indicating that treatment with gypenosides did not cause significant hepatotoxicity. However, unlike control mice, gypenoside-treated mice showed signs of tumor necrosis (Figure 6E). Furthermore, immunohistochemistry results revealed that the levels of Ki67, cPLA2, and CYP1A1 were reduced compared with those in the control group. The expression level of COX2 was significantly higher than that in the control group (Figure 6F). The results showed that gypenosides could inhibit tumor growth without hepatotoxicity *in vivo*. This mechanism suggests that gypenosides affect tumor growth by regulating the expression of cPLA2, CYP1A1, and COX2 in the arachidonic acid pathway.

## Discussion

The development of ccRCC is strongly linked to lipid metabolism (Lowrance et al., 2010). Lipid accumulation is currently considered to be an important marker of the aggressiveness of RCC, indicating that reprogramming of lipid metabolism may occur during the development of renal cancer (Capitanio et al., 2019). Lipid metabolism plays an important role in tumor cell proliferation and metastasis. Among the related pathways, arachidonic acid metabolism, sphingolipid metabolism, and steroid biosynthesis play a central role in the development of many diseases (Wang and Dubois, 2010; Dennis and Norris, 2015; Sonnweber et al., 2018). Arachidonic acid is an omega-6 polyunsaturated fatty acid whose metabolism-related enzymes and products participate in inflammation and regulate various cellular processes, including cell proliferation, angiogenesis, tumor invasion, metastasis, etc. (Yarla et al., 2016). Phospholipase A2 (PLA2s) is the initial enzyme of the AA metabolic pathway, which converts cell membrane-bound phospholipids into free fatty acids under various stimuli, mainly arachidonic acid and lysophospholipids (Dessen et al., 1999; Gijón and Leslie, 1999; Yarla et al., 2016). Of note, COX, LOX, and CYP 450 enzymes and their inhibitors are widely used to treat inflammation and cancer (Fishbein et al., 2021). Several prior studies have found that COX-2, LOX-1, and their inhibitors can reduce resistance and enhance sensitivity to chemotherapeutic drugs (Apaya et al., 2016). COX-2 and PGD2 have previously been identified as potential targets for the prevention and treatment of colon cancer (Wang and Dubois, 2010). However, COX-2 and CYP450 are also key enzymes that can stimulate the resolution of inflammation and produce pro-resolving mediators (SPMs), such as lipoxins (LXA4) and EETs (Wallace, 2006). Arachidonic acid metabolism products, including prostaglandins (PGs), leukotrienes (LTs), EETs, and HETEs play a role in inhibiting tumor cell apoptosis, stimulating angiogenesis, and enhancing cell proliferation and metastasis (Schneider and Pozzi, 2011; Chen and Wang, 2013). Thus, targeting lipid metabolism may be an effective treatment strategy for renal cell carcinoma. However, previous studies have focused on the treatment of inflammatory diseases, such as hepatitis or hyperlipidemia by regulating lipid metabolism (Li et al., 2020; Weng et al., 2021). Here, we demonstrate that the gypenosides Gyp L and Gyp LI could reduce the content of arachidonic acid in ccRCC cells by downregulating cPLA2, thereby inhibiting the growth of renal cancer. This observation promotes the possibility that gypenoside could significantly increase the sensitivity of cancers to cPLA2 inhibitors and could, thus, provide a new approach for the treatment of renal cancer. Although we demonstrated a decrease in AA following treatment, further work is needed to analyze the content of metabolites, such as EETs and PGE2 in cells and tumors treated with gypenosides.

Mitogen-activated protein kinase (MAPK) is an important transmitter, which functions to transmit signals from the cell surface to the nucleus *via* the phosphorylation of key protein targets following activation by different extracellular stimuli, including cytokines, cell stress, and cell adhesion. The continuous activation of the upstream MAPK kinase kinase (MAPKKK) and MAPK kinase (MAPKK) leads to the activation of MAPK (Donohoe et al., 2020). The MAPK pathway is also mediated by ERK, JNK, and p38 protein kinases (Johnson and Lapadat, 2002). The cascade of extracellular signal-regulated kinases ERK1 and ERK2 (ERK1/2) is closely related to cancer and is strongly involved in multiple tumor processes, including cell differentiation, cell senescence, and apoptosis *via* the phosphorylation of multiple target proteins (Deschênes-Simard et al., 2014). The dysregulation of the JNK pathway is also closely associated with cancer; this pathway is involved in various cellular processes, such as cell proliferation, survival, apoptosis, and inflammation (Hammouda et al., 2020). p38 plays a dual role in tumorigenesis, alternatively acting as a tumor suppressor and a tumor promoter (Martínez-Limón et al., 2020). Importantly, previous studies have demonstrated that p38 and p42/p44 MAPK are essential for ATPgammaS-induced COX-2 expression and PGE2 synthesis (Lin et al., 2009). Notably, ERK1/2 regulates PKC protein in a dependent and independent manner, and further mediates cPLA2 phosphorylation and AA release in astrocytes (Xu et al., 2002). We have demonstrated the efficacy of gypenosides in inducing apoptosis of renal cell carcinoma *via* activation of the PI3K/AKT/mTOR pathway (Liu et al., 2021). This fits with previous research, which has shown that gypenosides inhibit the proliferation of liver and esophageal cancer by regulating the MAPK pathway (Ma et al., 2019). However, the applications and mechanisms of action of gypenosides Gyp L and Gyp LI, which modulate the progression of renal cancer through the MAPK pathway, remain largely obscure.

**FIGURE 7:**
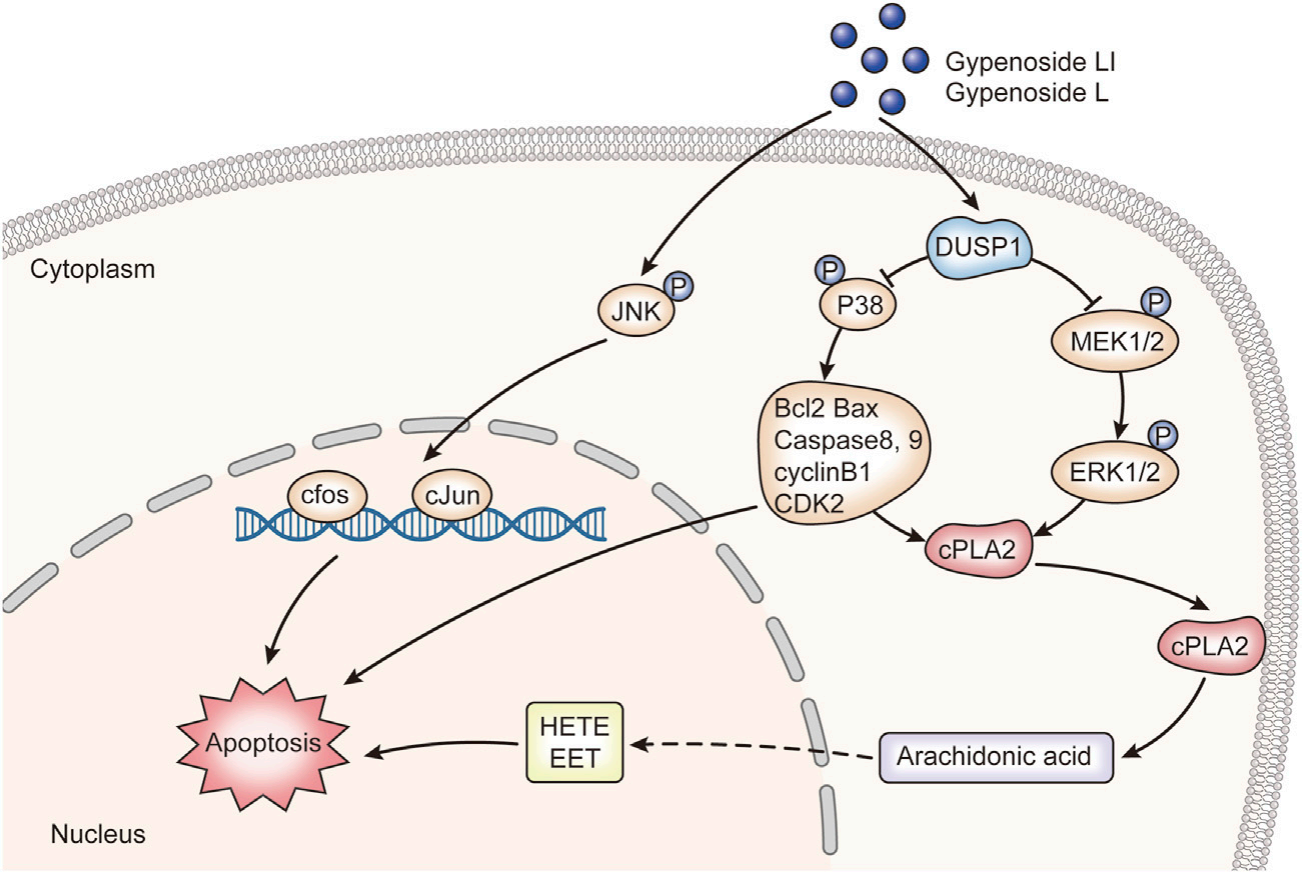
The molecular mechanism of gypenoside L and gypenoside LI inducing apoptosis in renal cell carcinoma.

In this study, the integration of network pharmacology and RNA-seq analysis revealed that gypenoside may inhibit the occurrence and development of renal cancer *via* action on the MAPK pathway. Here, we experimentally demonstrated that Gyp L and Gyp LI significantly inhibited the proliferation of 769-P and ACHN by upregulating DUSP1 and downregulating p-P38, p-MEK, and p-ERK. We also confirmed that Gyp L and Gyp LI induced apoptosis in ccRCC cells by upregulating p-JUN, p-c-Jun, and c-fos. We hypothesized that key genes in the MAPK pathway and in the metabolism of arachidonic acid regulate arachidonic acid levels in ccRCC cells and contribute to tumor growth (Figure 7). However, this hypothesis still requires experimental validation. How gypenosides regulate the metabolism of arachidonic acid through the MAPK pathway requires further investigation. Overall, the gypenosides, Gyp L, and Gyp LI may be safe and effective drugs for the treatment of ccRCC.

## Conclusion

In conclusion, the present study demonstrates that Gyp L and Gyp LI can both cap inhibit the proliferation of ccRCC cells by regulating key genes in the MAPK pathway and the metabolism of arachidonic acid. Gypenosides reduce the content of arachidonic acid by downregulating cPLA2 levels *in vivo* to inhibit tumor growth without inducing hepatotoxicity. Although further research is necessary, this study provides preliminary results to indicate that Gyp L and Gyp LI are promising drugs in the treatment of renal cancers, specifically ccRCC.

## Data Availability

The datasets presented in this study can be found in online repositories. The names of the repository/repositories and accession number(s) can be found below: https://www.ncbi.nlm.nih.gov/bioproject/PRJNA785289/.
